# Single-subject analyses of magnetoencephalographic evoked responses to the acoustic properties of affective non-verbal vocalizations

**DOI:** 10.3389/fnins.2014.00422

**Published:** 2014-12-22

**Authors:** Emilie Salvia, Patricia E. G. Bestelmeyer, Sonja A. Kotz, Guillaume A. Rousselet, Cyril R. Pernet, Joachim Gross, Pascal Belin

**Affiliations:** ^1^Centre for Cognitive Neuroimaging, Institute of Neuroscience and Psychology, University of GlasgowGlasgow, UK; ^2^Bangor Imaging Unit, School of Psychology, Bangor UniversityGwynedd, UK; ^3^School of Psychological Sciences, University of ManchesterManchester, UK; ^4^Max Planck Institute for Human Cognitive and Brain SciencesLeipzig, Germany; ^5^Brain Research Imaging Center, Division of Clinical Neurosciences, Western General Hospital, University of EdinburghEdinburgh, UK; ^6^Départment de Psychologie, Université de MontréalMontreal, Canada; ^7^Institut des Neurosciences de La Timone, UMR 7289, CNRS & Aix-Marseille UniversitéMarseille, France

**Keywords:** acoustics, ANVs, emotions, GLM, magneto-encephalography, single-subject, voice morphing

## Abstract

Magneto-encephalography (MEG) was used to examine the cerebral response to affective non-verbal vocalizations (ANVs) at the single-subject level. Stimuli consisted of non-verbal affect bursts from the Montreal Affective Voices morphed to parametrically vary acoustical structure and perceived emotional properties. Scalp magnetic fields were recorded in three participants while they performed a 3-alternative forced choice emotion categorization task (Anger, Fear, Pleasure). Each participant performed more than 6000 trials to allow single-subject level statistical analyses using a new toolbox which implements the general linear model (GLM) on stimulus-specific responses (LIMO-EEG). For each participant we estimated “simple” models [including just one affective regressor (Arousal or Valence)] as well as “combined” models (including acoustical regressors). Results from the “simple” models revealed in every participant the significant early effects (as early as ~100 ms after onset) of Valence and Arousal already reported at the group-level in previous work. However, the “combined” models showed that few effects of Arousal remained after removing the acoustically-explained variance, whereas significant effects of Valence remained especially at late stages. This study demonstrates (i) that single-subject analyses replicate the results observed at early stages by group-level studies and (ii) the feasibility of GLM-based analysis of MEG data. It also suggests that early modulation of MEG amplitude by affective stimuli partly reflects their acoustical properties.

## Introduction

Accurate recognition and interpretation of emotional states is crucial for social interaction. Humans communicate their feelings by verbal or non-verbal means such as body gestures, facial expressions, or affective non-verbal vocalizations (ANVs). Indeed, in addition to gender, age and other attributes, voices convey information about the speaker's emotional state (Belin et al., 2004, 2011; Schirmer and Kotz, 2006). Yet only few studies have investigated the emotional content conveyed by voices, particularly in the absence of speech (Morris et al., 1999; Fecteau et al., 2007; Sauter and Eimer, 2010; Kotz et al., 2013; Bestelmeyer et al., 2014).

Recent electrophysiological studies performing group-level analyses and using ANVs have suggested rapid processing of emotional information. Electro-encephalography (EEG) studies found evoked response differences between affective and neutral vocalizations as early as 100 ms (Jessen and Kotz, 2011; Liu et al., 2012) or 150 ms (Sauter and Eimer, 2010) after stimulus onset. The effect of emotion on the P200 evoked component is more equivocal: studies, using verbal (Paulmann and Kotz, 2008b; Paulmann et al., 2008; Schirmer et al., 2013) or non-verbal (Jessen and Kotz, 2011; Liu et al., 2012) affective stimuli showed that the P200 amplitude is either enhanced (Jessen and Kotz, 2011; Liu et al., 2012; Schirmer et al., 2013) or reduced (Paulmann and Kotz, 2008b; Paulmann et al., 2008) for arousing relative to neutral vocalizations. Compared to neutral stimuli, emotionally intense stimuli are generally associated with a larger Late Positive Potential (LPP) component (~400–600 ms) over the centro-parietal sensors (Keil et al., 2002; Schupp et al., 2006; Kanske and Kotz, 2007; Flaisch et al., 2008; Herbert et al., 2008; Olofsson et al., 2008; Pastor et al., 2008; Paulmann and Kotz, 2008a; Liu et al., 2012).

However, these effects are always reported at the level of entire groups of participants, leaving unclear whether they can be observed at the single-subject level. In addition, it is not entirely clear to what extent these early “emotional” effects are in fact driven by acoustics: sounds that vary in affective properties also tend to have different acoustical structures. Thus, Schirmer and Kotz (2006) reported that frequency and sound intensity modulate the amplitude of an evoked component that peaks ~100 ms following stimulus onset (i.e., N100). Other acoustical measures (e.g., mean and standard deviation (SD) of the fundamental frequency; mean and SD intensity, duration) have also been shown to partly explain cerebral activity in the temporal regions (i.e., Superior Temporal Sulcus and Superior Temporal Gyrus), occurring with a latency of ~200 ms (Grandjean et al., 2005; Schirmer and Kotz, 2006; Fecteau et al., 2007; Wiethoff et al., 2008; Leaver and Rauschecker, 2010; Kotz et al., 2013).

**Figure 1:**
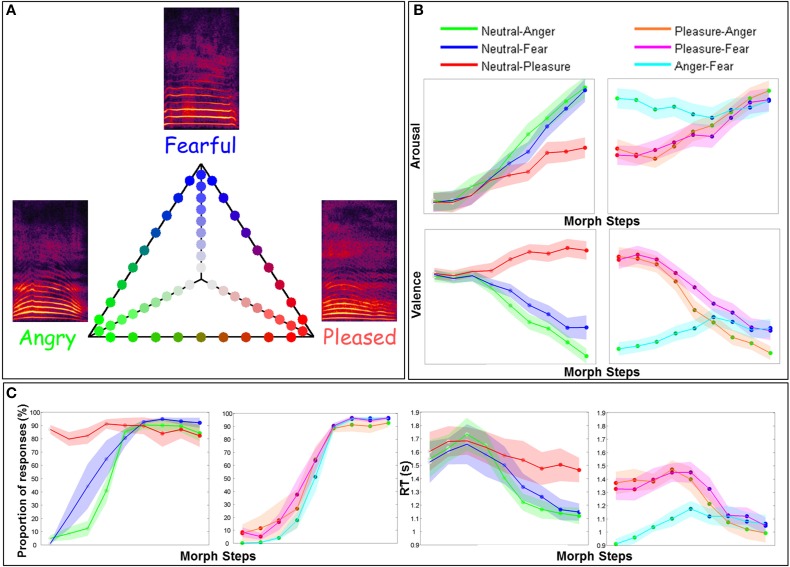
**Stimulus presentation, ratings, and behavioral results. (A)** Stimuli generated by morphing between non-verbal affective vocalizations expressing Anger, Fear, Pleasure or a Neutral expression using the vowel /a/. Two sets of 54 stimuli were used, one from a male speaker and one from a female speaker. The six continua (each of them including 9 stimuli), between either two emotions or a neutral expression and an emotion, are presented on the triangle. Each stimulus is represented by a specific color: Anger in green, Fear in blue, Pleasure in red and the Neutral expression in gray. **(B)** Ratings of perceived Arousal and Valence. On the left, ratings for continua between the Neutral expression and each of the three emotions are presented. Colored lines represent continua between the Neutral expression and either Anger in green, Fear in blue or Pleasure in red. On the right side of the panel, ratings for continua between two emotions are presented. Orange, mauve and cyan lines represent continua between Pleasure-Anger, Pleasure-Fear, and Anger-Fear, respectively. The x-axis represents the morph steps from 1 to 9 (9 stimuli per continuum) and the y-axis either the Arousal or the Valence from 2 to 7 (each stimulus was rated using a 9-points Likert scale). The mean is based on 25 (Arousal) or 24 (Valence) participants and two identities (male—female). Shades around each line represent the standard error of the mean. **(C)** These graphs represent the behavioral data from the three MEG participants. The two graphs on the left represent the results for the proportion of Anger, Fear or Pleasure responses and the two graphs on the right represent the results for the reaction time (RT). In the first graph on the left, green, blue, and red lines represent the proportion of Anger, Fear or Pleasure responses, for each stimulus, for continua between the Neutral expression and either Anger (Anger responses were considered), Fear (Fear responses), and Pleasure (Pleasure responses). In the second graph, cyan, orange, and mauve lines represent the proportion of responses for Anger-Fear (Fear responses were considered), Pleasure-Anger (Anger responses), and Pleasure-Fear (Fear responses) continua. The color code of these lines is exactly the same for the RT graphs. For both response accuracy and RT, the x-axis represents the morph steps, from 1 to 9. The y-axes extend from 0 to 100% (proportion of responses) for response accuracy and from 0.9 to 1.9 s for RT graphs. We computed a mean on data from three participants and two identities (male—female) and then computed the standard error represented by the shades around each line. For both (Panels **B,C**) the stimulus color code (i.e., color of the circles on each continuum line) is as in **(A)**.

Here we examined whether early effects of emotion parameters on MEG amplitude can be observed at the single-participant level. A small number of participants (*n* = 3) listening to a large number of stimuli (*n* > 6000) were included. Stimuli were based on the Montreal Affective Voices (MAVs, Belin et al., 2008), which consist of a validated database of ANVs with minimal interaction of emotional and linguistic content (Banse and Scherer, 1996; Schirmer and Kotz, 2006; Belin et al., 2008). Stimuli were parametrically manipulated using morphing (Figure 1), to create stimuli with wide variations in acoustical and emotional properties. Analyses were performed using a novel toolbox implementing the GLM for EEG/MEG data (LIMO EEG, Pernet et al., 2011), to examine how much of these early effects are explained by acoustic properties of the stimulus. We hypothesized that early effects of perceived Valence and Arousal could be observed at the single-subject level, but that these early components, modulated by low-level sound features (Gunji et al., 2003; Capilla et al., 2013), should be mainly sensitive to acoustic rather than perceptual characteristics.

## Materials and methods

### Participants

Three healthy volunteers took part in the experiment (2 males: 26 and 35 years old; 1 female: 21 years old). All participants reported normal hearing. They all provided informed written consent and received monetary compensation for their participation. The local ethics committee approved the study.

### Stimuli and task

Stimuli were generated by morphing between recordings of non-verbal affective vocalizations from the MAVs database (Belin et al., 2008) in which actors were instructed to produce emotional interjections using the vowel /a/. The vocalizations were produced by two different actors (i.e., one male and one female) each of them expressing anger, fear, pleasure as well as a neutral expression. Stimuli were generated by morphing between pairs of vocalizations from the same speaker to keep speaker identity constant within a continuum.

Voice morphing was performed using STRAIGHT (Kawahara and Matsui, 2003) in Matlab 2010a (Mathworks, Inc, Natick, USA). STRAIGHT performs an instantaneous pitch-adaptive spectral smoothing in each stimulus for separation of contributions to the voice signal arising from the glottal source vs. supra-laryngeal filtering. A voice stimulus is decomposed by STRAIGHT into five parameters: f0, frequency, time, spectro-temporal density, and aperiodicity that can be manipulated and combined across stimuli independently of one another. Time-frequency landmarks to be put in correspondence across voices during morphing were manually identified in each stimulus, and corresponded to the frequencies of the first three formants at onset and offset of phonation.

A continuum between vocalizations A and B consisted of 9 stimuli (6 continua per speaker—see Figure 1A), each containing a proportion of both A and B, progressing in acoustically equal 15% steps: 110%A/−10%B, 95%A/5%B, 80%A/20%B…20%A/80%B, 5%A/95%B, −10%A/110%B. The first and last stimuli of each continuum (−10%/110% and 110%/−10%) were slight caricatures of one emotion relative to the other. In total, 108 stimuli were used, i.e., 54 per identity. Two kinds of continua were produced: between Neutral and each of the three emotions (Neutral-Anger, Neutral-Fear, and Neutral-Pleasure) and between pairs of emotions (Anger-Fear, Pleasure-Anger, Pleasure-Fear—see Figure 1A). Stimulus duration was kept constant across emotions to the average duration of the original vocalizations (male voice: 1000 ms; female voice: 780 ms).

Stimuli were validated in a behavioral rating phase. Ninety-eight participants performed an online experiment where they were requested to rate either male or female stimuli on either perceived Arousal, i.e., the intensity of the emotion, or Valence, i.e., the degree of pleasantness (25 participants for Arousal and 24 participants for Valence, per identity). For Arousal, 10 men and 15 women performed the experiment for the female voice and 7 men and 18 women for the male voice. For Valence, 9 men and 15 women did the experiment for the female voice and 6 men and 18 women for the male voice. They rated each stimulus twice using a 9-points Likert scale (from 1 = very low perceived Arousal/Valence to 9 = very high perceived Arousal/Valence).

During scanning, participants performed a 3-alternative forced choice classification of the stimuli's perceived emotion: anger, fear or pleasure. Participants were instructed to respond as quickly as possible using a non-magnetic response pad. Stimuli were presented and responses recorded using the Psychtoolbox-3 (Brainard, 1997; Kleiner et al., 2007) in Matlab 2007. Stimuli were delivered binaurally using a custom-made sound pressure transducer with two 5-m long plastic tubes terminating in plastic insert earpieces. During sound stimulation, a black fixation cross was presented through a DLP projector (PT-D7700E-K, Panasonic) on a white background at the center of the projection screen. Participants were instructed to maintain their gaze at the fixation cross while listening to the sounds, which helped reduce movements as much as possible. Participants were scanned in 8 sessions of ~2.50 h each (~20 h of MEG experiment per subject). Each session consisted of 5 runs, each lasting ~12 min. Each run included three blocks, each of them made of 54 stimuli of either male or female vocalizations. All 54 stimuli in each block occurred without repetition. Half of the runs consisted of 2 male/1 female blocks and the other half was made up of 1 male/2 female blocks. At the end of the experiment, participants were exposed to 60 blocks of male vocalizations and to 60 blocks of female ones (i.e., each stimulus was repeated 60 times). In each block, the participants listened to randomized sequences of these morphs with a stimulus onset asynchrony (SOA) randomly selected between 3.5 and 4.5 s. Blocks were interleaved with resting periods of 20 s, where participants were requested to remain still and relaxed. Every two runs, unlimited time was given to rest outside of the magnetically shielded room. The overall experiment included in total 6480 trials (2 speakers ^*^54 stimuli ^*^60 repetitions). This large number of trials per participant allowed (i) performing subject-level analyses and (ii) an increased statistical power to dissociate acoustical from perceptual effects.

One-Way analyses of variance (ANOVA) were performed to test the effect of the factor “sessions” (810 trials per session, i.e., each stimulus was repeated either 7 or 8 times) on the reaction time (RT). Four different ANOVAs were performed, i.e., for each stimulus category (Anger, Fear, Pleasure) and one bringing together all of the stimuli.

### MEG recording

A 248-magnetometers whole-head MEG system (MAGNES® 3600 WH, 4-D Neuroimaging), confined in a magnetically shielded room, was used to record brain activity. MEG signal was acquired at a 1 kHz sampling rate. Before data acquisition, 5 coils were placed on the participants' head to allow accurate localization both at the beginning and the end of each run. These coils, as well as three fiducial points and the participants' head shape were digitized using a Polhemus system (Polhemus Inc.). During the recording, participants were seated in a reclining chair and supported their head against the back and top of the magnetometer. Participants were instructed to remain as still as possible and were also continuously monitored by a video camera. They were also asked to prevent blinking while the auditory stimuli were presented.

### MEG analysis

The analysis of MEG signal was performed using the FieldTrip software package (Oostenveld et al., 2011; http://www.ru.nl/fcdonders/fieldtrip/) as well as in-house Matlab code using functions predominantly taken from the LIMO EEG toolbox (Pernet et al., 2011) that implemented the framework of the GLM.

#### Pre-processing

The signal was epoched into trials of 1.5 s length (500 ms pre-stimulus) time-locked to stimulus onset. Noisy sensors were selected visually and then removed using the FieldTrip's *ft_rejectvisual* function. Signals recorded by the MEG reference sensors were used to reduce noise, as implemented in FieldTrip's *ft_denoise_pca* function. The signal was also down-sampled at 256 Hz and finally digitally low-pass filtered below 20 Hz and baseline corrected using the 500 ms pre-stimulus time window.

#### Event related fields (ERFs) analysis

**Figure 2:**
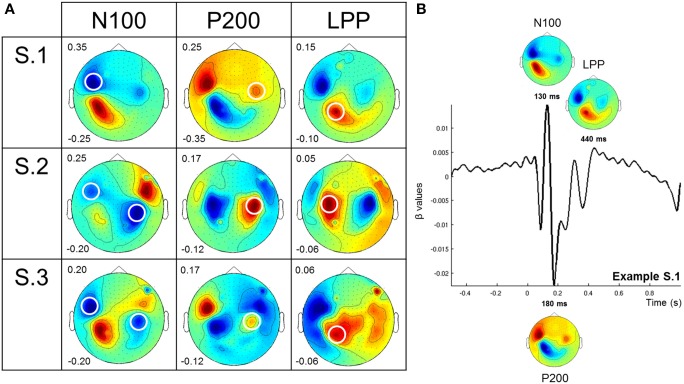
**ERF topographies. (A)**. Topographies from three main ERF components elicited by auditory stimuli (for all stimuli pooled together): the N100 peaking between 50 and 140 ms, the P200 peaking between 150 and 250 ms and the LPP peaking between 400 and 600 ms. Circled sensors are those showing a negative and a positive amplitude modulation for negative (N100) and positive (P200 and LPP) components, respectively. We particularly focused our attention on these sensors in this study. Numbers on the left of each topographic plot (i.e., for each ERF component and each participant) are the maximum (upper values) and the minimum (lower values) β-values. **(B)**. Example from S.1. This graph shows the baseline activity of the overall channels and especially shows the three peaks selected to plot the corresponding topographies: the N100 which show a minimum at 130 ms, the P200 at 180 ms, and the LPP at 440 ms. The N100, P200, and LPP topographies are located either above (N100 and LPP) or below (P200) their peaks.

We were interested in both early N100 and P200 ERF components, included in the pre-defined 50–140 ms (N100) and 150–250 ms (P200) temporal windows and in the LPP included in the 400–600 ms temporal window. The parameter estimates of the constant term of one of our “simple” GLM were used to plot the topographies of the baseline evoked response magnitude (i.e., the adjusted mean across all stimuli). We used Fieldtrip's *ft_singleplotER* function to first plot an average of the signal across sensors (see Figure 2B) and then to select the ERF peaks included in the three pre-defined temporal windows to get the corresponding topographies (Figure 2A). We report the peak latencies for each component in the Results section.

#### GLM analyses

One aim of this study was to assess the proportion of MEG signal variance accounted for by acoustical features, and whether correlations between ERF amplitudes and Valence/Arousal remain significant after removing that acoustically-explained variance. For this we used the framework of the General Linear Model (GLM), i.e., a novel approach to process MEG data. There are probably complex non-linear relations between acoustics and emotions but we simply wanted to eliminate basic, simple linear relations, e.g., louder means more arousing. Therefore, we only removed linear functions of acoustics.

Two models were estimated for both Arousal and Valence. The “simple” models included a single regressor (Arousal or Valence), whereas the “combined” models included two additional acoustical regressors to examine the influence of low-level acoustical parameters (Leaver and Rauschecker, 2010). The acoustical regressors corresponded to the first two components from a principal component analysis (PCA) of z-scored values from six acoustical features measured using Praat (Boersma and Weenink, 2009; www.praat.org). These six acoustical features included the mean and the SD of the fundamental frequency (f0) measured over the voiced portion (in Hz), the harmonic-to-noise ratio (HNR) of the voice parts (in dB) and the percentages of unvoiced frame, jitter and shimmer. The first two acoustical components from the PCA, included in the “combined” model explained 72% of the variance. Loadings of the different acoustical components are shown in Table 1. Both dimensions (i.e., Arousal/Valence) were highly correlated to the acoustical parameters. Correlations between Arousal and acoustical components 1 and 2 are −0.21 and −0.78, respectively. Correlations between Valence and acoustical components 1 and 2 are less important than those observed between Arousal and acoustics with values of 0.17 and 0.53, respectively. GLMs with highly inter-correlated continuous variables can be inaccurate to assess the effect of several continuous variables on the independent variable. Therefore, we orthogonalized the dimension considered in the model (either Arousal or Valence) against the subspace defined by the two acoustical regressors: thus Arousal and Valence parameter estimation correspond to variance not explained by acoustical parameters. Equally, as perceived Arousal and Valence are highly correlated, we included either Arousal or Valence as emotional dimension in separate models.

**Table 1:** **Loadings of the acoustical components**.

	**PC1**	**PC2**
Mean f0	0.56	−0.69
SD f0	−0.15	−0.85
HNR	0.97	−0.12
% unvoiced frame	−0.47	−0.33
Jitter	−0.86	−0.29
Shimmer	−0.82	0.03

*Correlation between principal components 1 (PC1) and 2 (PC2) and the six acoustical features entered in the PCA—mean and SD f0, HNR, % of unvoiced frame, jitter and shimmer*.

GLM analyses were performed using the LIMO EEG toolbox (Pernet et al., 2011). Parameters (β-values) were estimated at each sensor and time point independently, yielding a matrix of 248 (sensors) ^*^385 (time points, from −0.5 to 1 s post-stimulus in 3.9 ms steps) for each regressor. Similar sensor ^*^time point matrices were computed for R^2^, F and *p*-values for both the overall models and for each regressor (partial *F*-values).

Probability values were determined using a permutation approach for which trial labels were permuted one thousand times. Clustering functions were then used in both space and time to correct for multiple comparisons (*p* ≤ 0.05): first, independently at each sensor and for each permutation, *F*-values (for the Arousal or Valence regressor) reaching the *p* ≤ 0.05 threshold, were clustered in time. The sum of *F*-values inside each temporal cluster was computed and the maximum sums were kept. Then, the maximum sums across sensors were sorted to obtain a 0.95 percentile threshold to which actual *F*-values were compared (Pernet et al., 2011; Rousselet et al., 2011; Bieniek et al., 2012).

## Results

### Online study: behavioral ratings of the affective stimuli

Figure 1B shows the ratings of perceived Valence and Arousal collected in an online study for all stimuli (three Emotion-Emotion continua and three Emotion-Neutral continua). Variations in affective dimensions along the morphed continua are clearly apparent (Figure 1B).

### Categorization task

Figure 1C presents the behavioral results, i.e., the proportion of Anger, Fear or Pleasure responses and RT, for the three participants at the three-alternative forced choice categorization task performed during MEG recordings.

No significant effect of the factor “sessions” on the RT was observed [Anger: *F*_(7, 120)_ = 0.53, NS; Fear: *F*_(7, 120)_ = 1.52, NS; Pleasure: *F*_(7, 120)_ = 1.37, NS; All: *F*_(7, 376)_ = 1.91, NS]. Whatever the emotion expressed, neither improvement nor decrement of the performance along the sessions was noticed.

### Imaging results

#### Event related fields analysis

Figure 2A shows scalp topographies corresponding to the three main components (N100, P200, LPP) elicited by auditory stimuli (for all stimuli pooled together) in each subject. For the N100, peak latencies were: 129, 121, and 133 ms; for the P200: 176, 234, and 211 ms; and for the LPP: 437, 590, and 566 ms for participants S1–S3 respectively. Topographies were consistent across participants except for S1 who showed no N100 in the right hemisphere. For the N100, both left and right occipital sensors showed either inward (i.e., positive, red) or outward (i.e., negative, blue) directed fields. Contralateral sensors showed opposite polarities. For the P200, right occipital sensors showed mostly an inward directed field. Contralateral sensors showed an outward directed field. For the LPP, we observed especially an inward directed field over the left central-parietal sensors.

#### GLM analyses

Two different models were used: the “simple” model and the “combined” model. By comparing the significant results (*p* ≤ 0.05 after correcting for multiple comparisons) from both models, we assessed the remaining effect of affective dimensions on MEG signal variance after controlling for the influence of low-level acoustical features. This direct comparison was possible because the Arousal and Valence regressors were orthogonal to the acoustic regressors and therefore the amount of variance explained was additive.

**Figure 3:**
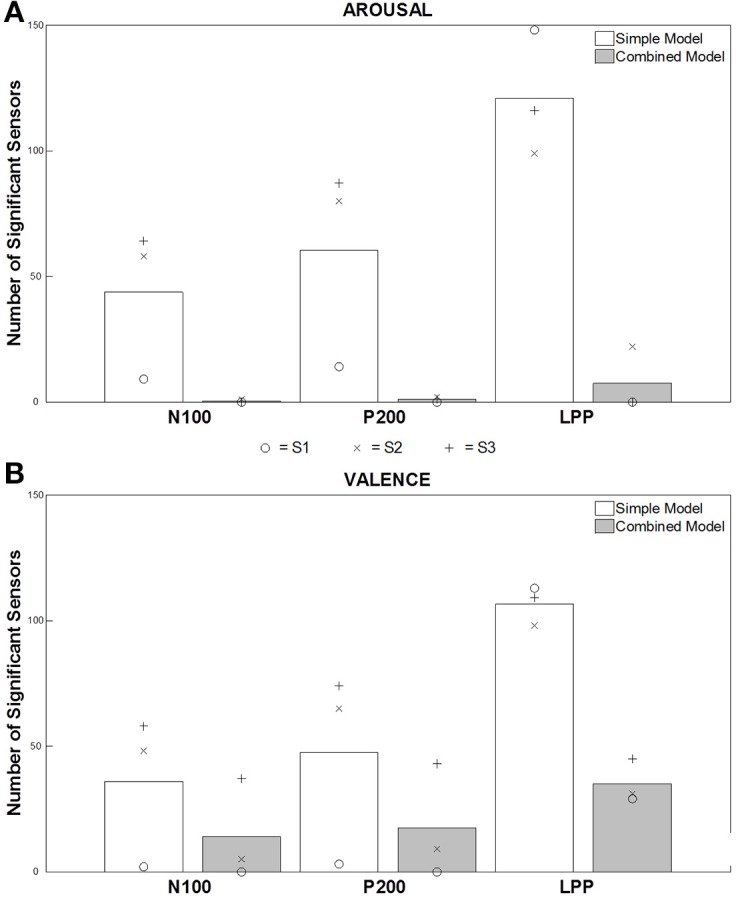
**Number of significant sensors for each ERF component and both models**. Each bar represents the average of the number of significant sensors (out of 248 sensors), computed on data from three participants, for the different conditions, i.e., N100-Simple Model, N100-Combined Model, P200-Simple Model, P200-Combined Model, LPP-Simple Model, LPP-Combined Model. White and gray bars present the results for each component for the “simple” model and the “combined” model, respectively. (**A,B**) present the results for the perceived Arousal and Valence, respectively. We attributed to each participant a symbol (o = S.1, x = S.2, and + = S.3) to show the results independently for each of them.

Figure 3 presents an average of the significant sensors, computed on data from the 3 participants, for each ERF component and both “simple” and “combined” models. For Arousal (Figure 3A), an important number of significant sensors were observed for both early (N100 and P200) and late (LPP) components before removing the acoustically explained variance (i.e., “simple” model). This number is very large for the LPP component. After controlling for the influence of the low-level acoustical characteristics on the signal variance (i.e., “combined” model), almost no significant sensors were observed for early components. Only few sensors remained significant for the LPP. For Valence (Figure 3B), we observed, as for Arousal, a large number of significant sensors for all components before mitigating the influence of acoustical parameters on the signal variance. A very large number of significant sensors for the LPP were as well observed. After controlling for the influence of acoustical parameters on signal variance, significant sensors were observed for early and late components, and more were observed for the LPP component.

**Figure 4:**
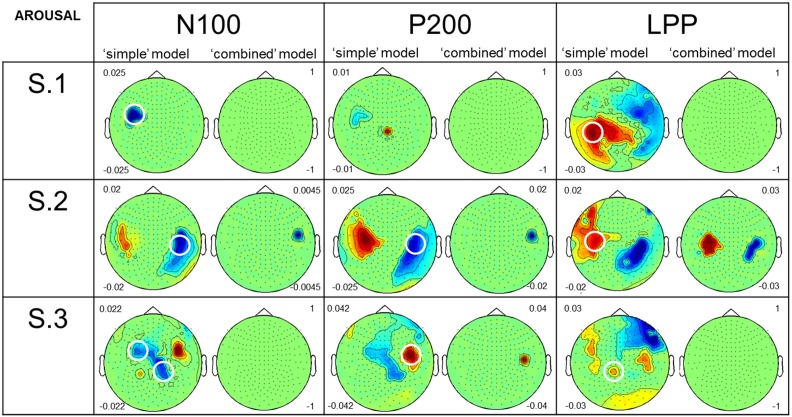
**Comparison of the results, for perceived Arousal, from the two GLM analyses**. The figure shows the results for the three participants, the N100, P200, and LPP, both models, and Arousal. Topographies present an average of β-values performed both on different time intervals of interest [i.e., 50–140 ms (N100), 150–250 ms (P200), 400–600 ms (LPP)] and on significant sensors and time points after correcting for multiple comparisons (*p* ≤ 0.05). Negative β-values (in blue) are equivalent to a decrease of ERF amplitude (or increase of negative ERF amplitude) caused by an increase in Arousal. It is the opposite for positive β-values (in red). Numbers on the left of the “simple” model topographies and on the right of the “combined” model ones are the maximum and the minimum β-values represented on the “simple” and the “combined” model topographies, respectively. We fixed these values at 1 (maximum) and −1 (minimum) when no significant effect was found. Circled sensors on the “simple” model topographies are similar or close to those circled in Figure 2A and showed large negative or positive correlations between MEG signal and perceived Arousal.

Figure 4 shows the results from both models (i.e., “simple” and “combined”) for Arousal. In this figure, scalp topographies illustrate β-values averaged over different time intervals of interest [i.e., 50–140 ms (N100), 150–250 ms (P200), 400–600 ms (LPP)]. These averages were performed on significant sensors and time points after correcting for multiple comparisons (*p* ≤ 0.05). Negative β-values (in blue) indicate a decrease of ERF amplitude (or an increase of negative ERF amplitude) with increased Arousal, and the opposite for positive β-values (in red). In the results reported below, we particularly focused our attention on sensors which are similar or close to those circled in the Figure 2A, i.e., showing baseline negative signal amplitude variation for the negative ERF (N100) and baseline positive signal amplitude variation for positive ERFs (P200 and LPP). For the N100 amplitude, results from the “simple” model showed large negative correlations with Arousal. Results from the “combined” model showed no (see S.1 and S.3) or very weak (see S.2) remaining correlations after statistically controlling for the influence of acoustical feature on signal variance. For the P200 amplitude, results from the “simple” model revealed large negative (see S.2) or positive (see S.3) correlations with Arousal. S.1 showed very weak correlations. For S.2 and S.3, results from the “combined” model showed weak remaining correlations. No remaining effect of Arousal on P200 amplitude was observed for S.1. For LPP amplitude, results from the “simple” model showed large positive correlations with Arousal for all the participants. For S.1 and S.3, results from the “combined” model presented no remaining correlation after adding acoustical parameters to the model. S.2, on the contrary, showed a large positive remaining correlation between Arousal and LPP amplitude.

**Figure 5:**
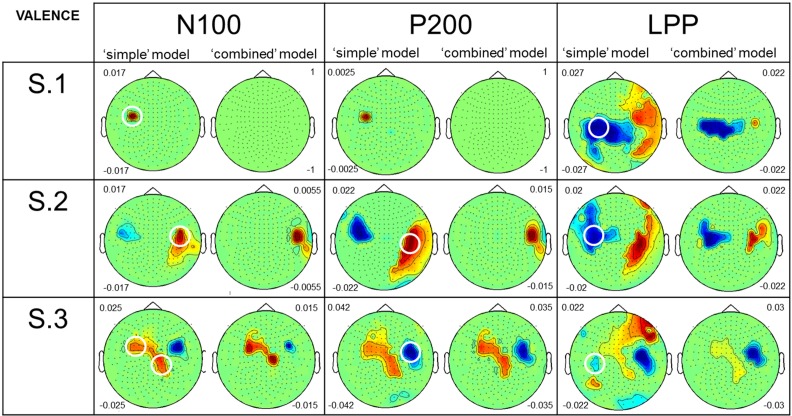
**Comparison of the results, for perceived Valence, from the two GLM analyses**. Caption as in Figure 4 although this figure presents results for perceived Valence.

Figure 5 presents results from both the “simple” and the “combined” models for Valence. As for perceived Arousal, we particularly focused our attention on sensors which are similar or close to those circled in the Figure 2A. For the N100 amplitude, in all participants, topographies from the “simple” model showed large positive correlations with Valence. For S.2 and S.3, results from the “combined” model revealed few remaining effects of Valence on MEG signal variance. S.1 showed no remaining effect. For P200 amplitude results from the “simple” model showed both positive (see S.2) and negative (see S.3) correlations with Valence. S.1 showed a weak correlation. Using the “combined” model, S.2 had only a weak remaining positive correlation. S.3 also showed remaining effects of Valence on signal variance. For S.1, no remaining correlation was observed. For LPP amplitude: results from the “simple” model showed negative correlations with Valence for all the participants. With the “combined” model, the three participants showed remaining effects of Valence on LPP amplitude.

***Signal variance explained by acoustical parameters vs. affective dimensions***. For each participant and each affective dimension (i.e., Arousal and Valence), R^2^ for both the “simple” and the “combined” models were computed. R^2^ values were averaged across all sensors. Therefore, the maximum R^2^ values are relatively weak (i.e., ~0.2). As two continuous variables (i.e., acoustics) were added in the “combined” model, the signal variance explained by this model is larger than those explained by the “simple” one. The partial correlation coefficients for each continuous variable included in the “combined” model, i.e., emotional dimensions (either Arousal or Valence) and acoustical parameters (i.e., average of the partial correlation coefficients of the two acoustical components) were computed as well. These coefficients present the impact of each continuous variable on the signal variance while the effects of the other continuous variables included in the model are controlled. These coefficients computed from the “combined” model revealed when affective dimensions and acoustical features explain most of the signal variance. For S.3, these coefficients revealed that both Valence and acoustical features explain the signal variance at both early and later stages. For S.1 and S.3, we observed no effect (S.1) or weak effects (S.3) of Arousal along all the time points after controlling for the influence of acoustical features. However, acoustical parameters explained the signal variance from early stages, from ~150 ms (S.3) or ~250 ms (S.1) after stimulus onset. The variance explained by these acoustical parameters lasted for a long time, until ~500 ms post-stimulus. For S.1 (for Valence) and S.2 (for both Arousal and Valence), the signal variance explained by acoustical parameters is earlier than that explained by emotions.

## Discussion

In this study, MEG was used to assess the temporal dynamics of emotional processing of ANVs. We employed an original approach in which we used (i) a small number of participants with a large number of stimuli each to perform single-participant analyses; (ii) voice morphing to generate stimuli with a large variation in acoustical and emotional properties; (iii) a massive univariate GLM to examine systematically the contribution of acoustics to early MEG effects. We confirm that early significant correlations between MEG amplitude and perceived emotional value shown by previous group-level analyses (Paulmann and Kotz, 2008b; Paulmann et al., 2008; Sauter and Eimer, 2010; Jessen and Kotz, 2011; Liu et al., 2012; Schirmer et al., 2013) can be observed at the single participant level. However, we show that these early effects are largely driven by acoustical variation and probably reflect the processing of acoustical rather than perceptual differences in the stimuli.

Our stimuli were validated based on online ratings of perceived Arousal and Valence by an independent group of participants. Wide variations in perceived Arousal and Valence were observed along the morphed continua (Figure 1B) and categorization performance for which our three participants (Figure 1C) produced the classical logistic curve with a steeper slope in the central, ambiguous part of the continua (Bestelmeyer et al., 2010). For continua between two emotions, RT was as expected: shorter for extreme stimuli and longer for ambiguous morphs. For continua between a neutral expression and an emotion, it was shorter for emotional and more easily recognizable stimuli, than for the neutral expression.

In this experiment, participants listened to a large number of stimuli (>6000 trials per participant) which should acquaint with these stimuli (habituation effect). We did not induce short-term habituation (i.e., when the same stimulus is presented in trains of stimuli), which could impact the ERF response amplitude (Woods and Elmasian, 1986; Polich, 1989; Rankin et al., 2009; Thompson, 2009). Nevertheless, long-term habituation (i.e., when stimuli are heard many times) may also have an effect on these responses. Habituation is defined as a behavioral response change that results from repeated stimulation (Rankin et al., 2009; Thompson, 2009). However, we did not notice significant RT differences across sessions though. Therefore, even if the habituation effect exists as in every, even shorter, experiment, we expected only a small habituation effect on MEG responses.

### “Simple” model: effects of valence and arousal without controlling for the influence of acoustics

Before removing the effect of acoustical parameters on signal variance, i.e., in the “simple” models, we observed large effects of emotional dimensions on early ERF amplitudes—N100 and P200 amplitudes in each of the three subjects. For perceived Arousal, we observed an increase of the negative N100 amplitude with increased Arousal. Further, we observed a positive correlation between perceived Valence and N100 amplitude, i.e., a decrease of the negative N100 amplitude with increased Valence. Previous group-level analyses, using non-verbal vocalizations without controlling for the impact of acoustics on signal variance (as in our “simple” model), also showed effects of emotions on the N100 component and especially smaller N100 amplitude for emotional compared to neutral stimuli (Jessen and Kotz, 2011, 2013; Liu et al., 2012). However, contrary to the outcomes of Liu et al. (2012), our results revealed that this early effect can not only be seen with Arousal but as well with Valence. For the P200 amplitude, significant results emerged, although not consistent across the three participants. We observed either a decrease (e.g., S.2) or an increase (e.g., S.3) of the P200 amplitude with increased Arousal. We also observed either an increase (e.g., S.2) or a decrease (e.g., S.3) of the P200 amplitude with increased Valence. Group-level analyses also highlighted similar inconsistencies: arousing vocalizations either increased the P200 amplitude (Sauter and Eimer, 2010; Jessen and Kotz, 2011, 2013; Liu et al., 2012; Schirmer et al., 2013) or decreased it (Paulmann and Kotz, 2008b; Paulmann et al., 2008). These findings, related to both N100 and P200 components, suggest an early rapid processing of affective information. Our study showed that these effects could also be observed at subject level.

In addition to these early effects, we observed late effects of emotion on signal variance. Results showed positive and negative correlations between LPP amplitude and Arousal and between LPP amplitude and Valence, respectively. Previous work, using mainly visual stimuli, has shown, at the group-level, that LPP amplitude is increased for arousing (Keil et al., 2002; Schupp et al., 2006; Flaisch et al., 2008; Olofsson et al., 2008; Pastor et al., 2008; Liu et al., 2012) and pleasant stimuli (Herbert et al., 2008). These correlations, observed between affective dimensions and LPP amplitude, are even greater than those observed between affective dimensions and early components (i.e., N100 and P200).

### “Combined” model: effects of valence and arousal after removing the acoustically-explained variance

The implementation of the GLM for electrophysiological data (LIMO EEG; Pernet et al., 2011) allowed removing signal variance explained by acoustical measures and therefore identifying the variance only explained by emotions. We observed, for both early and late components, fewer remaining effects for perceived Arousal than for perceived Valence after controlling for the influence of acoustical characteristics. This difference between the two emotional dimensions may be due to stronger correlations between Arousal and both acoustical components (−0.21 and −0.78) than those observed between Valence and these components (0.17 and 0.53). Variance explained by Arousal in the “simple” model may be mainly explained by acoustics conveying emotional information in the speaker's voice (Grandjean et al., 2005; Schirmer and Kotz, 2006; Fecteau et al., 2007; Wiethoff et al., 2008; Leaver and Rauschecker, 2010; Kotz et al., 2013; Bestelmeyer et al., 2014). This notion is supported by the partial correlation analysis, which revealed, especially in S.1 and S.3, that the signal variance is mostly driven by acoustics from early (~200 ms) to late (~500 ms after stimulus onset) stages with no or weak effects of Arousal.

For perceived Arousal, we reported only a remaining effect on LPP amplitude for the second participant. No or only weak other remaining effects (on all component amplitudes) were observed. For all participants, we also observed remaining late effects of perceived Valence on LPP amplitude. Therefore, while removing acoustically-explained variance, the remaining effects of emotional variables (especially Valence) are mostly observed at late stages (~400–600 ms). As few remaining early effects were observed, it seems that early effects are mainly driven by acoustics, in keeping with studies which performed group-level analyses suggesting that low-level sound features are processed at early latencies (Gunji et al., 2003; Capilla et al., 2013). However, results from the third subject also revealed strong early effects of Valence on both N100 and P200 amplitude, providing solid evidence of inter-individual variability in this effect.

## Conclusion

We used subject-level GLM analyses—a relatively novel approach to analyses MEG data—to compare the signal variance explained by emotional dimensions of the stimuli (perceived Arousal and Valence) both before and after statistically controlling for the influence of acoustical structure. We found that early effects of Arousal are in fact largely explained by variance in acoustical features, consistent with Schirmer and Kotz (2006) who noted that understanding vocal emotional messages requires analysing and integrating a variety of acoustical cues. In contrast however, perceived Valence contributed more strongly to variance in MEG amplitude independently of acoustics, particularly at later processing stages. These results are consistent with a processing of emotional vocalizations in different processing stages, which may include perceptual encoding, stimulus representation in working memory, and then an elaborate stimulus evaluation along affective dimensions (Schupp et al., 2006). Interestingly, these subject-level analyses also revealed a high degree of inter-individual variability across participants that is not generally acknowledged by group-level analyses. Indeed, this study, while using a small number of participants but with a large number of trials each, (i) manages to explain the divergences documented in the literature (from group-level analyses) which revealed inconsistent P200 results and therefore (ii) shows that each individual processes affective stimuli in different ways.

### Conflict of interest statement

The authors declare that the research was conducted in the absence of any commercial or financial relationships that could be construed as a potential conflict of interest.
